# Supplementary material for: Evans syndrome in children below 13 years of age – A nationwide population-based cohort study
Source: PLoS One. 2020 Apr 9;15(4):e0231284. doi: 10.1371/journal.pone.0231284 (PMC7145102; doi:10.1371/journal.pone.0231284)
Supplement: Attachment — Submitted filename: Review for PONE-D-20-01354.docx [file pone.0231284.s001.docx]

The manuscript PONE-D-20-01354 titled ‘***Evans syndrome in children below13 years of age-a nationwide population-based cohort study***” describes the incidence, prevalence and hazard ratio for death of children with Evans syndrome in Denmark. Since this is one of the very few registry-based studies on this very rare autoimmune disorder, it deserves consideration of publication. Of course, most, if not all of the described results have previously been shown by others. For example, it is well-known that Evans syndrome has worse prognosis and increased mortality compared to AIHA or ITP alone. Although the authors should be congratulated for the study, Denmark is a very small country with just 5,6 million people. A Scandinavia-based study that would include patients from Denmark, Sweden, Norway and Finland (estimated population of 26 million people), would add strength to the study’s conclusions, since Evans syndrome is so rare in childhood that e.g., only 2 deaths were recorded over the entire study period. Moreover, several points of the manuscript need clarifications and revisions.

First, the definition of secondary Evans syndrome that was used is extremely problematic. One year of follow-up after Evans’ syndrome diagnosis is completely inadequate to exclude with confidence cases of secondary disease. Although the authors refer to this limitation in the discussion, this is done at the wrong place (lines 125-127). A dedicated paragraph with the limitations of the study should be added just prior to the concluding paragraph of the discussion.

Second, the authors fail to discuss one of the main findings of the study, i.e., the fact that Evans syndrome in Denmark is more common in boys (2 to 1 ratio) like it is in France (reference No. 6). The authors should elaborate on this in the discussion, since indeed Evans syndrome appears to be more common in males during childhood but more common in females during adulthood (as it is the case with other autoimmune diseases). In addition, potential explanations for this sex difference by age should be offered.

Third, the authors should elaborate on the potential reasons for the increased incidence and prevalence of Evans syndrome with time, i.e., they should give some plausible explanations for the almost 2.5 times higher incidence and prevalence of Evans syndrome in 2015 compared to 1990.

Fourth, it would be interesting to study the year in which the 4 splenectomies for patients with Evans syndrome were performed. In the 1990s splenectomy was likely more commonly employed for Evans syndrome , AIHA or ITP compared to 2015, but the relevant data should be provided.

Fifth, more references are required, and a more in-depth discussion is required. Finally, there are few typos and missing words throughout the manuscript. For example, in the abstract (line 20), the word register should be replaced with the word **registry**, since the described study is a registry-based not a register-based study. Also, in line 64 the word between is missing (All patients with ES below 13 years of age diagnosed **between** 1981-2015 were included in analyses). Also, in lines 138-139, the word is should be added: Despite of this, we managed to identify only 21 patients emphasizing that pediatric ES **is** a very rare entity.
