# Supplementary material for: Evans syndrome in children below 13 years of age – A nationwide population-based cohort study
Source: PLoS One. 2020 Apr 9;15(4):e0231284. doi: 10.1371/journal.pone.0231284 (PMC7145102; doi:10.1371/journal.pone.0231284)
Supplement: Attachment — Submitted filename: Rebutal Letter & Response to Reviewers.docx [file pone.0231284.s002.docx]

**David Meyre**, Academic Editor	8^th^ of March, 2020
PLOS ONE

Regarding manuscript PONE-D-20-01354 entitled: *Evans syndrome in children below 13 years of age – a nationwide population-based cohort study*

*Dear David Meyre,*

Thank you very much for your response to our manuscript. We appreciate the constructive comments and the opportunity to resubmit a revised manuscript to your journal.

We have revised the manuscript and incorporated as much relevant information as possible. We enclose point-by-point answers to the concerns raised below.

We hope that we have addressed your concerns satisfactorily, but remain of course open to further revision.

Kind regards,

Nikolaj Mannering, M.D., Senior Registrar in Hematology

Department of Haematology

Odense University Hospital

Kloevervaenget 6, Entrance 93, 12th floor

DK – 5000 Odense C

Denmark

Telephone: +45 21450372

E-mail: [nikolaj.mannering2@rsyd.dk](mailto:nikolaj.mannering2@rsyd.dk)

**Academic Editor/Journal requirements**:

1. When submitting your revision, we need you to address these additional requirements.
Please ensure that your manuscript meets PLOS ONE's style requirements, including those for file naming. The PLOS ONE style templates can be found at
http://www.journals.plos.org/plosone/s/file?id=wjVg/PLOSOne_formatting_sample_main_body.pdf and http://www.journals.plos.org/plosone/s/file?id=ba62/PLOSOne_formatting_sample_title_authors_affiliations.pdf

Response:

*Done*

2. In the ethics statement in the manuscript and in the online submission form, please provide additional information about the patient records used in your retrospective study. Specifically, please ensure that you have discussed whether all data were fully anonymized before you accessed them and/or whether the IRB or ethics committee waived the requirement for informed consent. If patients provided informed written consent to have data from their medical records used in research, please include this information.

Response:

*All data used in this retrospective study were completely anonymized, and no detailed patient information e.g. social security numbers or detailed journal information, were available. Permission from The Danish Ethics Committee was therefore not necessary, neither were any written consents from any of the participating patients.*

3. We note that you have indicated that data from this study are available upon request. PLOS only allows data to be available upon request if there are legal or ethical restrictions on sharing data publicly. For information on unacceptable data access restrictions, please see http://journals.plos.org/plosone/s/data-availability#loc-unacceptable-data-access-restrictions.
**In your revised cover letter, please address the following prompts**:
a) If there are ethical or legal restrictions on sharing a de-identified data set, please explain them in detail (e.g., data contain potentially identifying or sensitive patient information) and who has imposed them (e.g., an ethics committee). Please also provide contact information for a data access committee, ethics committee, or other institutional body to which data requests may be sent.

Response:

*The data used in this study are extracted from the Danish Patients Registries, and are available at a server at Statistics Denmark. Public sharing of national health data are not allowed according to Danish law, but may be accessed upon request through application to The Danish Data Protection Agency as well as the specific registries.*

*Since the study is a registry-based study, no specific or unique data on the patients (e.g. medical files, blood samples etc.) have been available during the study. Pure registry-based research does not require Ethics Committee approval, according to Danish law.*

b) If there are no restrictions, please upload the minimal anonymized data set necessary to replicate your study findings as either Supporting Information files or to a stable, public repository and provide us with the relevant URLs, DOIs, or accession numbers. Please see http://www.bmj.com/content/340/bmj.c181.long for guidelines on how to de-identify and prepare clinical data for publication. For a list of acceptable repositories, please see http://journals.plos.org/plosone/s/data-availability#loc-recommended-repositories.
We will update your Data Availability statement on your behalf to reflect the information you provide.

Response:

*Not relevant – please see response under “a)”*

4. Thank you for stating the following in the Competing Interests/Financial Disclosure section:

This project was part of a PhD fellowship (DLH), which was supported by the University of Southern Denmark (SDUSF-2015-202-(459)) https://www.sdu.dk/en/, The Region of Southern Denmark (16/13496) https://www.regionsyddanmark.dk/wm228983, and study grants from Alexion Pharma Nordic AB https://alexion.com/SelectCountry/Denmark, The A.P. Møller and Chastine Mc-Kinney Møller Foundation (17-L-0334) https://www.apmollerfonde.dk/ansoegning/laegefonden/, and Novartis Healthcare https://www.novartis.com/.
None of the authors have any other disclosures.
The funders had no role in study design, data collection and analysis, decision to publish, or preparation of the manuscript.

We note that you received funding from a commercial source: Alexion Pharma Nordic and Novartis Healthcare.

Please provide an amended Competing Interests Statement that explicitly states this commercial funder, along with any other [relevant declarations](http://www.plosone.org/static/competing.action) relating to employment, consultancy, patents, products in development, marketed products, etc.

Within this Competing Interests Statement, please confirm that this does not alter your adherence to all PLOS ONE policies on sharing data and materials by including the following statement: "This does not alter our adherence to PLOS ONE policies on sharing data and materials.” (as detailed online in our guide for authors <http://journals.plos.org/plosone/s/competing-interests>).  If there are restrictions on sharing of data and/or materials, please state these. Please note that we cannot proceed with consideration of your article until this information has been declared.

**Please include your amended Competing Interests Statement within your cover letter.** We will change the online submission form on your behalf.

Please know it is PLOS ONE policy for corresponding authors to declare, on behalf of all authors, all potential competing interests for the purposes of transparency. PLOS defines a competing interest as anything that interferes with, or could reasonably be perceived as interfering with, the full and objective presentation, peer review, editorial decision-making, or publication of research or non-research articles submitted to one of the journals. Competing interests can be financial or non-financial, professional, or personal. Competing interests can arise in relationship to an organization or another person. Please follow this link to our website for more details on competing interests: <http://journals.plos.org/plosone/s/competing-interests>

Response:

*Amended Competing Interests Statement: The project was funded by Alexion Pharma Nordic and Novartis Healthcare. The funders had no role in study design, data collection and analysis, decision to publish, or preparation of the manuscript. None of the authors have any financial interests of any kind in any of the companies, employment in e.g. Advisory Boards or consultancy work. None of the authors are currently participating in any kind of drug development related to any of the companies. This does not alter our adherence to PLOS ONE policies on sharing data and materials.*

**Reviewer #1**:

The paper is very interesting and I compliment the authors for such a thorough investigation and long-term follow-up.
I have some suggestions for improvement, all of which cover minor issues.

1) Abstract, “The epidemiology and prognosis of Evans syndrome in patients above 13 years of age has recently been investigated”; this is not reported in the following text, and there is no clear reference; I guess that the authors refer to reference 6; anyway, the authors do not compare their present results to the previous ones.

Response:

*Thank you, for the overall positive comment. No references are allowed in the abstract section, but the relevant reference is no. 7 by “Hansen et al., AJH, 2019”. In our Discussion section (page 10, line 172-176) we address this issue, comparing the study results with similar results in adults:*

“…Our survival analyses indicate that particular in AIHA, mortality and the occurrence of ES is increased, most pronounced in secondary ES cases. These results are in line with our previous study of adults with ES where particularly patients with secondary ES had a dismal prognosis [1]. In a French study from 2009 on adults, a corresponding analysis in contrast showed no difference in survival between patients with primary and secondary ES[2]”

2) Introduction, “Here we assess both frequency and prognosis in Danish children below 13 years of age with ES”; why below 13 years? Please detail such a choice.

Response:

*In a recent work by us on Evans syndrome, we defined adults as persons aged >12 years of age and included these in our analyses. A cut-off of 18 years of age could have been used as well in this study, but since data from patients between 13-18 years of age were already published in our previous study, we refrained from including these data in a publication again and therefore chose to set the cut-off below 13 years of age.*


3) Methods, “ES was defined as the cumulative registration of ITP and AIHA in the same patient”: it should be emphasized that the diagnosis of ITP and AIHA does not necessarily be simultaneous.

Response:

*We agree that it is very important to convey this message to the readers. Currently we address this in the Discussion section, but we have now also changed the text in Methods section so it now reads (page 4, line 63-65):*

*“…*In short, ES was defined as the cumulative registration of ITP and AIHA in the same patient. The registration of the diagnoses could be sequential (85.7% of the cases) or simultaneous (14.3% of the cases).”

4) Results, “in the period 1980-2016” In the Methods it was reported 1981-2015.

Response:

*We apologize for these discrepancies. The data were collected from the period 1981-2015. The period 1980-2016 is a typo, and this has been corrected accordingly in the revised manuscript.*

5) Results, “Splenectomy was performed in four (19%) patients” should be postponed; I would rather report the epidemiological data first, and the clinical data thereafter.

Response:

*We agree with this. The sentence has been postponed in the Results section (page 8, line 114-115), so the text now reads:*

*“…*Splenectomy was performed in four (19%) patients. Splenectomy rates were higher in patients with ES, both compared to the general population, and to patients with AIHA or ITP (Table).”

6) Table, in the first column the “(%)” can be misleading, since what the other columns report, within parentheses, are the confidence intervals

Response:

*We agree that this can be misleading. Currently we present data to comply with the general style requirements for PLOSONE. In order to ease the interpretation we have added the following text to the table heading:*

“…(please note that all intervals in parenthesis following numbers in the table are 95% confidence intervals)”

7) Results, “four respectively one patient died”: I did not catch the meaning; furthermore, when reporting deaths, the authors report only percentages; it would help the comprehension to have absolute numbers

Response:

*We agree that this sentence can be ambiguous. It refers to the 4 deaths among patients with AIHA and the 1 death among patients with ITP. We report percentages in the table and absolute numbers in the text. However, to clarify the results we have changed the paragraph (page 8, line 119-123) so it now reads:*

“…During the study, two patients with ES died, both within the first year following diagnosis. One of the patients suffered from secondary ES. The causes of death in patients with ES were registered as bleeding or malignancy.

In the AIHA comparison group four patients died, and in the ITP comparison group one patient died. Causes of death in these patients were registered as haemolysis or were not recorded.”

8) Discussion: the only therapy included in the whole paper is splenectomy, that has been considered an indicator of need for “stronger” treatments; however, the paper would have a deeper and sharper meaning if the severity of the disease and the challenge of the therapy would have been discussed.

Response:

*We agree that such data are of great interest. However, our data are registry-based, and detailed results from treatment, blood tests etc. are unfortunately not available to us. However, in order to address severity of the Evans syndrome in general we have added a short paragraph discussing the challenging aspects of ES treatment in children (page 10-11, line 181-187). It reads:*

“…Treatment of ES in children is generally challenging [3, 4]. The cornerstone in front-line treatment is steroids[4], especially in cases where AIHA is clinically dominant while IVIG can be preferred in cases with pronounced thrombocytopenia[3]. However, many patients need second-line treatments (e.g. rituximab and mycophenolate mofetil)[5], and there is a general lack of randomized clinical trials investigating optimal treatment strategies. The heterogeneity of mechanisms underlying ES including secondary causes, requires that the diagnostic work-up becomes even more thorough in order to individualize treatment [3, 6].”

9) Also, due to the rarity of the condition, some recent and insightful papers should be included in the reference list: Miano M et al. Mycophenolate mofetil for the treatment of children with immune thrombocytopenia and Evans syndrome. A retrospective data review from the Italian association of paediatric haematology/oncology. Br J Haematol. 2016 Nov;175(3):490-495
Miano M. How I manage Evans Syndrome and AIHA cases in children. Br J Haematol. 2016 Feb;172(4):524-34.
Ladogana S et al.Diagnosis and management of newly diagnosed childhood autoimmune haemolytic anaemia. Recommendations from the Red Cell Study Group of the Paediatric Haemato-Oncology Italian Association. Blood Transfus 2017; 15(3):259-67

Response:

*We agree that the paper would improve from extending the discussion and referencing the suggested papers. We have extended the discussion and addressed the issues raised by the reviewers, and provide more references including some of the proposed.*

**Reviewer #2**:

The manuscript PONE-D-20-01354 titled ‘Evans syndrome in children below 13 years of age-a nationwide population-based cohort study” describes the incidence, prevalence and hazard ratio for death of children with Evans syndrome in Denmark. Since this is one of the very few registry-based studies on this very rare autoimmune disorder, it deserves consideration of publication. Of course, most, if not all of the described results have previously been shown by others. For example, it is well-known that Evans syndrome has worse prognosis and increased mortality compared to AIHA or ITP alone.

Response:

*Thank you, for the overall positive evaluation. We agree that some of our results are in line with in previous studies. However, to our knowledge, no previous study has published estimates of ES prevalence, and only one has published estimates of ES incidence (Aladjidi et al., 2015).*

*We generally find a lack of population-based studies in children with this rare disorder. Most of the previous cohorts comprises fewer patients than ours and focuses mostly on pathophysiology and/or treatment. We believe that our paper can contribute with additional knowledge on ES in children, and emphasize the need for more focus on this rare and severe disease”.*

Although the authors should be congratulated for the study, Denmark is a very small country with just 5,6 million people. A Scandinavia-based study that would include patients from Denmark, Sweden, Norway and Finland (estimated population of 26 million people), would add strength to the study’s conclusions, since Evans syndrome is so rare in childhood that e.g., only 2 deaths were recorded over the entire study period.

Response:

*We agree that more patients from our Scandinavian neighbors would indeed have added strength to our conclusions. However, we chose to do this as a national study because data from our Scandinavian colleagues were not available to us at the time the study was carried out.*

**First**, the definition of secondary Evans syndrome that was used is extremely problematic. One year of follow-up after Evans’ syndrome diagnosis is completely inadequate to exclude with confidence cases of secondary disease. Although the authors refer to this limitation in the discussion, this is done at the wrong place (lines 125-127). A dedicated paragraph with the limitations of the study should be added just prior to the concluding paragraph of the discussion.

Response:

*We agree that an underlying secondary defining diagnosis in ES may occur later than one year after ES diagnosis in clinical care. We assign the primary vs. secondary ES subtype from the time point (+ one year) where our ES diagnosis criteria were fulfilled and do not use later records of secondary defining diagnosis in order to avoid conditioning on future events. This is a standard approach in registry studies since conditioning on future events causes biased survival analysis [e.g. Karim-ME et al* [*Am J Epidemiol*](https://www.ncbi.nlm.nih.gov/pmc/articles/PMC4983651/)*. 2016; 184(4): 325–335].*

*In our dedicated paragraph (page 12, line 212-224) on the limitations of the study we have discussed this shortcoming in our results:*

“…We define secondary ES as the occurrence of associated diagnosis before or up to 1 year after diagnosis. However, secondary cases of ES may first be apparent up to several years after ES diagnosis [6]. In another study, diagnoses associated with ES were included from 6 years before up to 15 years after and was found to be mostly infections, immunodeficiency syndromes, and autoimmune diseases [6]. Possible underlying genetically conditioned immune disorders and differential diagnoses to ES (e.g. autoimmune lymphoproliferative syndrome (ALPS) and common variable immunodefiency (CVID)), and the potential under diagnosis of these, especially in the beginning of our study period, could contribute to ES being misclassified as primary instead of secondary [6]. This could indicate that with longer follow-up, the proportion of ES patients with underlying associated diseases increases.”

**Second**, the authors fail to discuss one of the main findings of the study, i.e., the fact that Evans syndrome in Denmark is more common in boys (2 to 1 ratio) like it is in France (reference No. 6). The authors should elaborate on this in the discussion, since indeed Evans syndrome appears to be more common in males during childhood but more common in females during adulthood (as it is the case with other autoimmune diseases). In addition, potential explanations for this sex difference by age should be offered.

Response:

*We agree that this finding is intriguing. We are not aware of any known causes in the literature for this early sex difference, but provide a discussion of this phenomenon in the new dedicated paragraph (page 9, line 140-151) addressing this issue below:*

“…We found that ES was more predominant in boys compared to girls with a sex ratio of 2:1. This is in contrast to the general distribution of autoimmune diseases in adulthood, where females are more predominant [7], and also to ES in adults where the overall sex ratio is 1:1[1]. The mechanism behind this phenomenon is unknown. A possible explanation to this could be differences in the pathophysiogical mechanisms preceding ES in children and adults respectively. Viral infections often precedes ES in young children[4], while adult patients may suffer from underlying haematological cancer (e.g. CLL[8]).

The reasons for the male predominance in very young patients with ES remain elusive. Genetically conditioned sex-differences, resulting in a more sensitive and responsive immune system, with a more profound release of cytokines (e.g. interferon-gamma[9]) following a viral infection may contribute to this [10].

Of note, ITP among boys below 5 years of age has a similar sex ratio of 2:1 as in ES [11].

**Third**, the authors should elaborate on the potential reasons for the increased incidence and prevalence of Evans syndrome with time, i.e., they should give some plausible explanations for the almost 2.5 times higher incidence and prevalence of Evans syndrome in 2015 compared to 1990.

Response:

*We agree that this finding is intriguing. However, unfortunately we do not have a definite answer to this finding. We note that a generally increased incidence of autoimmune disease has been seen in other autoimmune disorders [Rose NR, 2016] and also advances in diagnostics may contribute. We have extended the paragraph (page 11, line 190-202), providing more possible explanations to this question. It now reads:*

“…During our study period both ES incidence and prevalence increased although results are based on small numbers, and ES frequency measures are imprecise. The reasons for increasing frequencies remain elusive. They may, however, reflect advances in diagnostic technologies, as well as an increased awareness on ES and its severity from clinicians.

A general increase in incidence of autoimmune diseases have been observed over the last decades, which may also influence the ES frequency in children [12]. Explanations to this general increase probably includes both genetic and environmental contributing factors [13]. Environmental factors such as pesticides and solvents, and antibiotics interfering with microbiotic flora in the gut, are believed to contribute significantly to the increase in autoimmune diseases [13, 14]. Others suggest that air pollution causes oxidative stress and interference with the immune system, leading to activation and dysregulation of B-cells and the production of auto-antibodies [15]. These factors, combined with increasing urbanization and hence some environmental exposures in the Western industrialized world may elevate the autoimmune disease frequencies [7]. ”

**Fourth**, it would be interesting to study the year in which the 4 splenectomies for patients with Evans syndrome were performed. In the 1990s splenectomy was likely more commonly employed for Evans syndrome, AIHA or ITP compared to 2015, but the relevant data should be provided.

Response:

*We agree that data on this are very interesting, and provide them in the Results (page 8, line 115-117) and Discussion (page 11, line 204-209) section below. However, we cannot provide more detailed results from small subgroups in health databases. This is due to Danish privacy regulations and regulations at statistics Denmark.*

Results

“…Of the four patients receiving splenectomy, three had the procedure done before the year 2000. Furthermore, all patients had the procedure done no more than 5 years after the Evans diagnosis”

Discussion

“…Most splenectomies were performed before the year 2000. This is probably due to limited medical treatments options in this period (e.g. rituximab), and corresponds with splenectomy being a more frequent treatment early in our study period [16]. However, our data are based on few numbers of patients. Splenectomy is usually reserved as a 3^rd^ or later line therapy in pediatric AIHA, ITP or ES, and with achieved complete remission rates and response durations greatly depending on disease with the poorest responses in ES [3, 16]”

**Fifth**, *more references* are required, and a more *in-depth discussion* is required.

Response:

*We have extended the discussion section, addressing the issues raised by the reviewers, and added more references in addition to this*

**Sixth**, there are few typos and missing words throughout the manuscript. For example, in the abstract (line 20), the word register should be replaced with the word registry, since the described study is a registry-based not a register-based study. Also, in line 64 the word between is missing (All patients with ES below 13 years of age diagnosed between 1981-2015 were included in analyses). Also, in lines 138-139, the word is should be added: Despite of this, we managed to identify only 21 patients emphasizing that pediatric ES is a very rare entity.

Response:

*Thank you for noticing these typos. We have corrected all accordingly.*

1.	Hansen DL, Moller S, Andersen K, Gaist D, Frederiksen H. Evans syndrome in adults - incidence, prevalence, and survival in a nationwide cohort. Am J Hematol. 2019. Epub 2019/07/12. doi: 10.1002/ajh.25574. PubMed PMID: 31292991.

2.	Michel M, Chanet V, Dechartres A, Morin AS, Piette JC, Cirasino L, et al. The spectrum of Evans syndrome in adults: new insight into the disease based on the analysis of 68 cases. Blood. 2009;114(15):3167-72. Epub 2009/07/30. doi: 10.1182/blood-2009-04-215368. PubMed PMID: 19638626.

3.	Mantadakis E, Farmaki E. Natural History, Pathogenesis, and Treatment of Evans Syndrome in Children. J Pediatr Hematol Oncol. 2017;39(6):413-9. Epub 2017/06/28. doi: 10.1097/MPH.0000000000000897. PubMed PMID: 28654461.

4.	Miano M. How I manage Evans Syndrome and AIHA cases in children. Br J Haematol. 2016;172(4):524-34. Epub 2015/12/03. doi: 10.1111/bjh.13866. PubMed PMID: 26625877.

5.	Miano M, Ramenghi U, Russo G, Rubert L, Barone A, Tucci F, et al. Mycophenolate mofetil for the treatment of children with immune thrombocytopenia and Evans syndrome. A retrospective data review from the Italian association of paediatric haematology/oncology. Br J Haematol. 2016;175(3):490-5. Epub 2016/10/28. doi: 10.1111/bjh.14261. PubMed PMID: 27447678.

6.	Aladjidi N, Fernandes H, Leblanc T, Vareliette A, Rieux-Laucat F, Bertrand Y, et al. Evans Syndrome in Children: Long-Term Outcome in a Prospective French National Observational Cohort. Frontiers in Pediatrics. 2015;3:79. Epub 2015/10/21. doi: 10.3389/fped.2015.00079. PubMed PMID: 26484337; PubMed Central PMCID: PMCPMC4586429.

7.	Rose NR. Prediction and Prevention of Autoimmune Disease in the 21st Century: A Review and Preview. Am J Epidemiol. 2016;183(5):403-6. Epub 2016/02/19. doi: 10.1093/aje/kwv292. PubMed PMID: 26888748.

8.	Jaime-Perez JC, Aguilar-Calderon PE, Salazar-Cavazos L, Gomez-Almaguer D. Evans syndrome: clinical perspectives, biological insights and treatment modalities. J Blood Med. 2018;9:171-84. Epub 2018/10/24. doi: 10.2147/JBM.S176144. PubMed PMID: 30349415; PubMed Central PMCID: PMCPMC6190623.

9.	Norton A, Roberts I. Management of Evans syndrome. Br J Haematol. 2006;132(2):125-37. Epub 2006/01/10. doi: 10.1111/j.1365-2141.2005.05809.x. PubMed PMID: 16398647.

10.	Uekert SJ, Akan G, Evans MD, Li Z, Roberg K, Tisler C, et al. Sex-related differences in immune development and the expression of atopy in early childhood. J Allergy Clin Immunol. 2006;118(6):1375-81. Epub 2006/12/13. doi: 10.1016/j.jaci.2006.09.008. PubMed PMID: 17157669.

11.	Yong M, Schoonen WM, Li L, Kanas G, Coalson J, Mowat F, et al. Epidemiology of paediatric immune thrombocytopenia in the General Practice Research Database. Br J Haematol. 2010;149(6):855-64. Epub 2010/04/10. doi: 10.1111/j.1365-2141.2010.08176.x. PubMed PMID: 20377590.

12.	Bach JF. The effect of infections on susceptibility to autoimmune and allergic diseases. N Engl J Med. 2002;347(12):911-20. Epub 2002/09/20. doi: 10.1056/NEJMra020100. PubMed PMID: 12239261.

13.	Khan MF, Wang H. Environmental Exposures and Autoimmune Diseases: Contribution of Gut Microbiome. Front Immunol. 2019;10:3094. Epub 2020/01/31. doi: 10.3389/fimmu.2019.03094. PubMed PMID: 31998327; PubMed Central PMCID: PMCPMC6970196.

14.	Vojdani A, Pollard KM, Campbell AW. Environmental triggers and autoimmunity. Autoimmune Dis. 2014;2014:798029. Epub 2015/01/23. doi: 10.1155/2014/798029. PubMed PMID: 25610638; PubMed Central PMCID: PMCPMC4290643.

15.	Zhao CN, Xu Z, Wu GC, Mao YM, Liu LN, Qian W, et al. Emerging role of air pollution in autoimmune diseases. Autoimmun Rev. 2019;18(6):607-14. Epub 2019/04/09. doi: 10.1016/j.autrev.2018.12.010. PubMed PMID: 30959217.

16.	Mathew P, Chen G, Wang W. Evans syndrome: results of a national survey. J Pediatr Hematol Oncol. 1997;19(5):433-7. Epub 1997/11/05. PubMed PMID: 9329465.
